# Increased levels of anti-dsDNA antibodies in immune complexes before treatment with belimumab associate with clinical response in patients with systemic lupus erythematosus

**DOI:** 10.1186/s13075-019-2056-y

**Published:** 2019-11-29

**Authors:** Azita Sohrabian, Ioannis Parodis, Nellie Carlströmer-Berthén, Martina Frodlund, Andreas Jönsen, Agneta Zickert, Christopher Sjöwall, Anders A. Bengtsson, Iva Gunnarsson, Johan Rönnelid

**Affiliations:** 1 Department of Immunology, Genetics and Pathology, Uppsala University, Rudbeck Laboratory, SE-75185 Uppsala, Sweden; 2 Rheumatology Unit, Department of Medicine, Karolinska Institutet, Stockholm, Sweden; 3 Rheumatology, Karolinska University Hospital, Stockholm, Sweden; 4 Rheumatology/AIR, Department of Clinical and Experimental Medicine, Linköping University, Linköping, Sweden; 5 Section of Rheumatology, Department of Clinical Sciences, Lund University, Lund, Sweden

**Keywords:** Systemic lupus erythematosus, Belimumab, Anti-nuclear autoantibodies, Anti-double-stranded DNA, Immune complexes, Therapy response

## Abstract

**Introduction:**

Immune complexes are of importance in systemic lupus erythematosus pathogenesis, and autoantibodies are believed to participate in immune complex formation. Quantification of autoantibody levels in circulating IC might be of prognostic value.

**Methods:**

A C1q-binding-eluting technique was applied to purify immune complexes from 55 belimumab-treated systemic lupus erythematosus patients during a 24-month follow-up. Autoantibodies in serum and in solubilized immune complexes were quantified using addressable laser bead immunoassay. We investigated whether levels of autoantibodies in immune complexes associate with disease activity and response to belimumab treatment.

**Results:**

High baseline anti-double-stranded DNA and anti-histone levels in immune complexes associated with attainment of zero scores in clinical systemic lupus erythematosus disease activity index 2000 during the 24-month follow-up (*p* = 0.003 and *p* = 0.048, respectively). Low complement levels associated with high serum anti-double-stranded DNA and anti-ribosomal P levels (*p* = 0.003 and *p* = 0.008, respectively) and high anti-double-stranded DNA (*p* = 0.002) but not anti-ribosomal P levels in immune complexes. Anti-SSA/SSB serum levels were lower in patients attaining lupus low disease activity state at month 6; these associations were stronger for corresponding immune complex levels. Serum levels of most autoantibodies had declined at month 3, whereas autoantibody levels in immune complexes, except for anti-double-stranded DNA, showed a more gradual decline over 1–2 years. Serum anti-double-stranded DNA levels decreased in all patients irrespective of systemic lupus erythematosus disease activity index 2000=0 attainment, whereas immune complex levels decreased only in achievers.

**Conclusion:**

Immune complex levels of autoantibodies against double-stranded DNA and the SSA/SSB complex show more specific associations with treatment outcome compared with serum levels in belimumab-treated systemic lupus erythematosus patients. Characterization of autoantibody content in circulating immune complexes could prove useful in treatment evaluation in systemic lupus erythematosus and other immune complex-associated diseases.

## Introduction

Systemic lupus erythematosus (SLE) is an autoimmune disease with heterogeneous expression, ranging from mild symptoms limited to the skin and joints to more severe manifestations such as renal involvement, severe cytopenia, or central nervous system disease [1]. SLE is characterized by a loss of immunologic tolerance, excessive formation of circulating anti-nuclear antibodies (ANA), and inflammatory involvement of multiple organs [1–5]. Autoantibodies are often presumed to participate in pathogenesis via formation of circulating and tissue-bound immune complexes (IC) [6–11].

Antibodies that recognize and bind to double-stranded (ds)DNA are serological hallmarks of both classification and disease activity in SLE [12]. The molecular characteristics of anti-dsDNA are heterogenous, with diverse relation to disease activity and severity. High-avidity anti-dsDNA antibodies are believed to be actively involved in the pathogenesis of lupus nephritis; however, low-avidity antibodies are most frequently associated with cerebral involvement [13–16]. Many SLE patients with a clinically quiescent disease may still show significant levels of anti-dsDNA in the circulation, suggesting that not all anti-dsDNA autoantibodies are pathogenic [17–21]. Pathogenicity is associated to the ability to activate complement, engage Fc receptors, and exert co-reactivity with non-DNA antigens [22, 23].

The mechanisms underlying deposition of IC and autoantibodies and subsequent tissue injury remain controversial. Either in situ binding of circulating autoantibodies to autoantigens planted in tissues [8, 24] or deposition of pre-formed circulating IC in tissues, notably mesangial matrix of the kidney [6, 10], may lead to classical complement activation and eventually tissue damage and organ failure. If specific autoantibodies like anti-dsDNA participate in the formation of circulating IC which later sequester in target organs, then quantification of the autoantibodies residing in circulating IC might be a valuable prognostic biomarker.

Belimumab, a recombinant human IgG_1_-λ monoclonal antibody against B lymphocyte stimulator (BLyS, also known as BAFF), is a biologic agent approved for the treatment of patients with active SLE despite ongoing standard of care therapy [25–27]. Treatment with belimumab decrease circulating anti-dsDNA levels [25, 28, 29]. There are to our knowledge no published studies on changes in levels of other autoantibodies or the role of IC or autoantibodies in IC in belimumab-treated SLE patients.

Several studies have reported difficulties in identification and quantification of autoantibodies in circulating IC. As long as antigen-binding sites are occupied in IC, specific antibodies will not bind in immunoassays. Anti-dsDNA in IC might be physically masked by anti-U1RNP or other antigen-antibody systems constituting major components of IC [30–32]. We have developed a bead-based assay where isolation of IC on magnetic beads is followed by a two-step elution procedure, during which IC are freed from the beads and (auto)antibodies are freed from corresponding (auto)antigens. This technique allows the quantification of multiple autoantibodies in circulating IC as we recently did in a work evaluating individual anti-citrullinated peptide antibodies (ACPA) in IC from patients with rheumatoid arthritis [33]. Here, we applied this assay to samples from SLE patients treated with belimumab in a real-life clinical setting, in order to evaluate whether quantification of autoantibodies in IC yields additional information in terms of disease activity and treatment response compared to conventional serum autoantibody measurements [29].

## Materials and methods

### Study patients and controls

A total of 55 patients with moderately active SLE despite standard of care therapy from three tertiary referral centers in Sweden at the Karolinska (*n* = 30), Lund (*n* = 18), and Linköping (*n* = 7) university hospitals, who initiated belimumab treatment were enrolled in this prospective observational study. All patients fulfilled the 1982 American College of Rheumatology and/or the Systemic Lupus International Collaborating Clinics (SLICC) classification criteria of SLE [34, 35]. After initiation of belimumab therapy, the patients were followed for a maximum of 24 months. Serum samples were obtained from 55, 41, 35, 30, and 22 patients at baseline after 3, 6, 12, and 24 months, respectively, and stored at − 80 °C until analysis. A more detailed description of the study criteria has been published previously [29]. Patient baseline characteristics are presented in Table 1.

**Table 1:** Baseline characteristics based on the 55 investigated SLE patients

Characteristic	
Female participants, *n* (%)	50 (90.9)
Ethnicity	
Caucasian*, n* (%)	52 (94.5)
African/African American, *n* (%)	3 (5.5)
Age (years), median (IQR)	41.2 (30.6–50.4)
SLE disease duration (years), median (IQR)	7.8 (4.3–14.2)
SLEDAI-2K, median (IQR)	8.0 (4.0–14.0)
C3 (g/L), median (IQR)	0.81 (0.62–1.02)
C4 (g/L), median (IQR)	0.11 (0.06–0.19)
Number of DMARDs tested until baseline*, median (IQR)	2 (1–3)
Number of DMARDs at baseline*, median (IQR)	1 (0–1)
Azathioprine, *n* (%)	18 (32.7)
Mycophenolate mofetil/sodium^†^, *n* (%)	10 (18.2)
Methotrexate, *n* (%)	8 (14.5)
Cyclosporine, *n* (%)	2 (3.6)
Antimalarial agents at baseline, *n* (%)	41 (74.5)
Prednisone equivalent dose at baseline (mg/day), median (IQR)	10.0 (8.4–12.9)
Reason for belimumab	
Mucocutaneous manifestations, *n* (%)	26 (47.3)
Musculoskeletal manifestations, *n* (%)	25 (45.5)
Hematological manifestations, *n* (%)	10 (18.2)
Lupus nephritis, *n* (%)	7 (12.7)
Neuropsychiatric SLE, *n* (%)	4 (7.3)
Serositis, *n* (%)	3 (5.5)
Constitutional symptoms^#^, *n* (%)	2 (3.6)

*Excluding antimalarial agents

^†^Mycophenolate mofetil (*n* = 9) and mycophenolate sodium (*n* = 1)

^#^Fatigue

*SLE* systemic lupus erythematosus, *SLEDAI-2K* systemic lupus erythematosus disease activity index 2000, *DMARDs* disease-modifying antirheumatic drugs, *IQR* interquartile range

Surveillance items included the SLE disease activity index 2000 (SLEDAI-2K) [36]. We used serum anti-dsDNA data centrally analyzed in Uppsala for this study to calculate SLEDAI-2 K at all time points. Treatment response was assessed using three different definitions: clinical (c)SLEDAI-2K=0 (a modification of SLEDAI-2K where complement levels and anti-dsDNA positivity are excluded) [37], attainment of Lupus Low Disease Activity State (LLDAS) [38], and the SLE responder index 4 (SRI-4) [25, 26, 28]. Details were described previously [29].

As method controls, sera from 20 healthy blood donors from Uppsala University Hospital were investigated for autoantibodies in sera and IC.

Written informed consent was obtained from all patients, and oral consent from the blood donor controls. The study was performed in compliance with the Helsinki Declaration, and the study protocol was approved by the regional ethics review boards in Stockholm, Lund, Linköping, and Uppsala.

### Capturing and isolation of circulating IC

Purification of IC from sera was conducted according to a previously described technique established in our laboratory [33]. In brief, purified human C1q (Quidel, San Diego, CA, USA) was attached to magnetic tosyl-activated microparticles (Dynabeads® M-280; Life Technologies, Carlsbad, CA, USA) according to recommendations by the manufacturer for activation of amine groups. Ten microliters of C1q beads was incubated with 10 μL serum and 30 μL PBS-0.05% Tween-1% BSA for 1.5 h on a microplate shaker (600 rpm) at 37 °C. The C1q-bound IC were sequentially eluted from C1q beads in two sequential steps utilizing 50 μL 0.1 M glycine-HCl, pH 2.5 followed by 100 μL freshly prepared 25% methanol, pH 11.5. The second elution step has previously been shown to allow freeing of antibodies from corresponding antigen with preservation of antigen specificity [32]. IC eluates that were not assayed the same day were stored at − 80 °C. A full description and validation of the method was published recently [33].

### Autoantibody detection

The levels of antibodies against nuclear antigens (dsDNA, histone, ribosomal P antigen, PCNA, SSA-Ro52, SSA-Ro60, SSB-La, Sm, U1RNP, and the Sm-U1RNP complex) in serum and in solubilized IC were determined with addressable laser bead immunoassay (ALBIA) applying Connective Profile FIDIS™ (Theradiag, Marne La Vallee, France) and according to descriptions by the manufacturer, with a minor modification in the acquisition of digital data from the ALBIA equipment to obtain readouts in the low measurement range for IC level quantification. IC eluates were diluted corresponding to dilution of the initial serum and incubated with fluorescent-labeled microsphere reagent for 1 h on a shaker at RT. Antibody specificities were detected utilizing a phycoerythrin-labeled anti-human IgG conjugate. The levels of antibodies in serum and corresponding IC fractions were expressed in arbitrary units per millilter (AU/mL) except for anti-dsDNA that was expressed in international units per millilter (IU/mL). Data were evaluated using Solinium software (Theradiag).

Serum concentrations of total amounts of C1q-binding circulating IC (CIC) were measured by Quanta Lite® ELISA (INOVA Diagnostics, San Diego, CA, USA), in accordance with instructions by the manufacturer. Age- and sex-matched population-based non-SLE controls from the Karolinska SLE cohort (*n* = 316) served as CIC controls.

Total immunoglobulin G (IgG) in serum and IC were determined using an in-house ELISA, also used in earlier studies [32, 39]. Samples were diluted with PBS-Tween and incubated with solid phase goat anti-human IgG (purified F(ab′)2, Fcγ specific 109-006-098, Jackson IR, USA). The bound IgG was identified using alkaline-phosphatase-conjugated goat anti-human IgG (purified F(ab′)2, Fcγ specific 109-056-098, Jackson). Total IgG concentrations were determined using a serially diluted serum with known immunoglobulin concentration as standard.

Complement factors C3 (reference range 0.67–1.29 g/L) and C4 (0.13–0.32 g/L) were determined using nephelometry.

### Statistical analyses

The non-parametric Mann-Whitney *U* test was employed to compare the levels of antibodies with regard to clinical features. Correlations were assessed using the Spearman’s rank correlation coefficient test. For comparisons across groups, the Kruskal Wallis test, and, for pairwise comparisons between baseline and follow-up, the Wilcoxon signed rank test were used.

As our approach of quantitating autoantibodies in solubilized IC is new, we did not know which way to express these data would be most informative in a clinical setting. Therefore, besides measurement of levels in solubilized IC, fractions of specific autoantibodies were also expressed as percentages (%) of levels in solubilized IC compared with levels obtained with conventional serum measurement in the same samples, or as enrichment of specific autoantibodies in IC where levels in IC and serum had been normalized to the total IgG levels in each compartment, according to the formula

$$ \left(\mathrm{autoantibody}\ \mathrm{level}\ \mathrm{in}\ \mathrm{IC}/\mathrm{total}\ \mathrm{IgG}\ \mathrm{in}\ \mathrm{IC}\right)/\left(\mathrm{level}\ \mathrm{of}\ \mathrm{autoantibody}\ \mathrm{in}\ \mathrm{serum}/\mathrm{total}\ \mathrm{IgG}\ \mathrm{in}\ \mathrm{serum}\right) $$

The statistical analyses were performed using JMP version 11 (SAS Institute, Cary, NC, USA). *p* values < 0.05 were considered statistically significant.

## Results

### Autoantibody levels in serum and IC

**Fig. 1:**
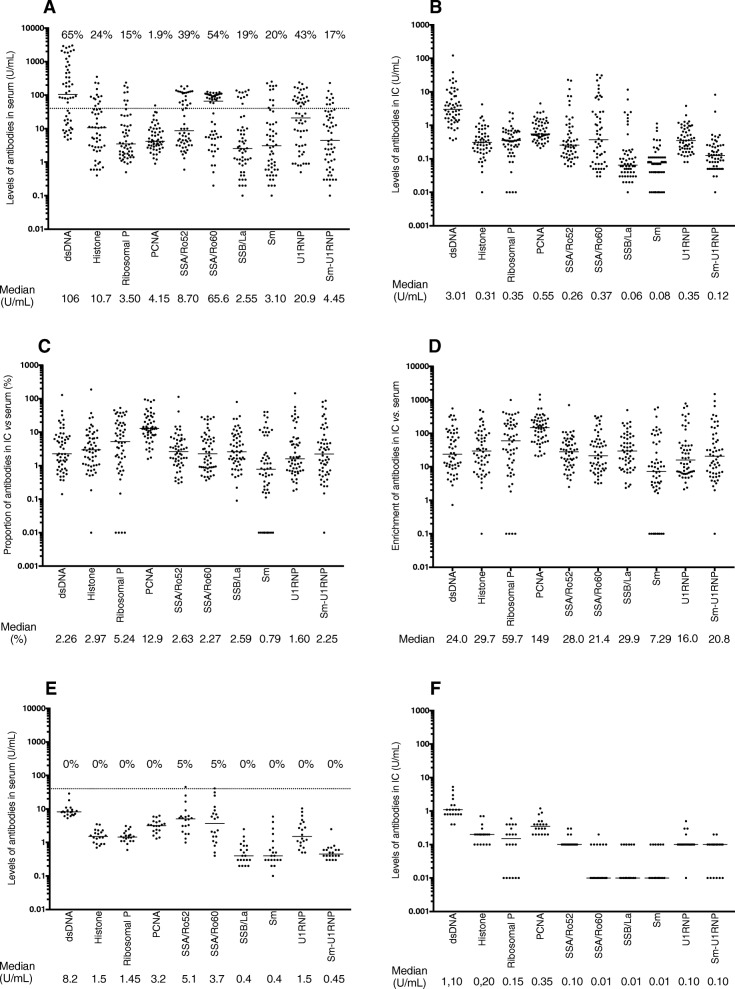
Distribution and levels of autoantibodies in sera (**a**, **e**) and corresponding solubilized IC fractions (**b**, **f**) for patients (**a**, **b**) and 20 healthy controls (**e**, **f**). Levels are presented in arbitrary units (AU/mL) for all antibodies except for anti-dsDNA, which is presented in international units (IU/mL). The ratio between autoantibody levels in IC and conventional measurement in sera among the patients are presented as **c** percentage in IC as compared to sera and **d** as enrichment after correction for total IgG concentrations in IC and sera, respectively. Median levels are illustrated as horizontal solid lines and the corresponding values are stated below each panel. The horizontal dotted line in **a** and **e** shows the clinically recommended cutoff values for autoantibodies in serum (40 U/mL). **a**, **e** The prevalence (%) of specific autoantibodies in serum is presented above each antibody. Values below the measurement ranges are depicted as 0.1 for sera and 0.01 in IC. Ribo P ribosomal P

Antibodies against dsDNA, SSA/Ro60, and U1RNP were detected in 65%, 54%, and 43% of the serum samples, whereas the corresponding proportions for other specificities were lower (Fig. 1a). Patterns of autoantibody distributions in solubilized IC (Fig. 1b) followed roughly the ones found in sera, with median IC levels ranging between 0.8 and 13% of the corresponding serum levels (Fig. 1c). When we normalized IC levels either as percentages compared to serum (Fig. 1c) or as antibody enrichments in IC (Fig. 1d), there were obvious differences in IC accumulation for different autoantibody specificities (*p* < 0.0001 for both approaches). All autoantibodies were enriched in IC compared to sera, with 7.3 to 149 times more autoantibodies in IC after correction for total IgG levels (Fig. 1d). Two autoantibody specificities deviated from remaining specificities: anti-PCNA was more enriched and anti-Sm was less enriched in IC compared to other autoantibodies (Fig. 1c, d; Table 2). Autoantibody levels in 20 healthy control sera and corresponding solubilized IC fractions were substantially lower (Fig. 1e, f).

**Table 2:** Difference in accumulation in immune complexes of different autoantibodies

Percentages in IC compared to those in sera	dsDNA, 2.26%	Histone, 2.97%	Rib P, 5.24%	PCNA, 12.9%	SSA/Ro52, 2.63%	SSA/Ro60, 2.27%	SSB/La, 2.59%	Sm, 0.788%	U1RNP, 1.60%
Histone, 2.97%	0.789								
Ribosomal P, 5.24%	0.103	0.115							
PCNA, 12.9%	*< 0.001*	*< 0.001*	*0.001*						
SSA/Ro5, 2.63%	0.706	0.537	0.031	*< 0.001*					
SSA/Ro60, 2.27%	0.636	0.405	0.040	*< 0.001*	0.835				
SSB/La, 2.59%	0.912	0.851	0.082	*< 0.001*	0.708	0.533			
Sm, 0.788%	*0.002*	*0.001*	*< 0.001*	*< 0.001*	*< 0.001*	*0.004*	*0.001*		
U1RNP, 1.60%	0.263	0.166	*0.027*	*< 0.001*	0.330	0.507	0.196	0.009	
Sm-U1RNP, 2.25%	0.446	0.409	0.061	*< 0.001*	0.630	0.733	0.454	0.006	0.794
Enrichment	dsDNA, 24.0	Histone, 29.7	Rib P, 59.7	PCNA, 149	SSA/Ro,52 28.0	SSA/Ro,60 21.4	SSB/La, 29.9	Sm, 7.29	U1RNP, 16.0
Histone, 29.7	0.895								
Ribosomal P, 59.7	0.153	0.155							
PCNA, 149	*< 0.001*	*< 0.001*	*0.002*						
SSA/Ro5, 28.0	0.738	0.541	*0.040*	*< 0.001*					
SSA/Ro60, 21.4	0.595	0.517	0.064	*< 0.001*	0.733				
SSB/La, 29.9	0.978	0.876	0.103	*< 0.001*	0.719	0.578			
Sm, 7.29	*< 0.001*	*< 0.001*	*< 0.001*	*< 0.001*	*< 0.001*	*< 0.001*	*< 0.001*		
U1RNP, 16.0	0.292	0.211	*0.040*	*< 0.001*	0.381	0.168	0.192	0.005	
Sm-U1RNP, 20.8	0.430	0.326	0.060	*< 0.001*	0.638	0.362	0.395	0.003	0.860

Results are shown for the percentage of levels in IC as compared to conventional measurement in sera in the upper part and as enrichment after correction for total IgG concentrations in IC and sera in the lower part. Median values for the percentage in IC as compared to that in serum and enrichment factors are presented below each autoantibody name in the upper and lower parts, respectively. Differences between individual autoantibody specificities concerning percentage in IC (upper part) and enrichment factors (lower part) were compared pairwise with the Wilcoxon signed rank test, and significant differences are depicted in italics. The corresponding distributions are shown in Fig. 1c, d. Significant *p* values are presented in italics. *Rib P* ribosomal P

**Fig. 2:**
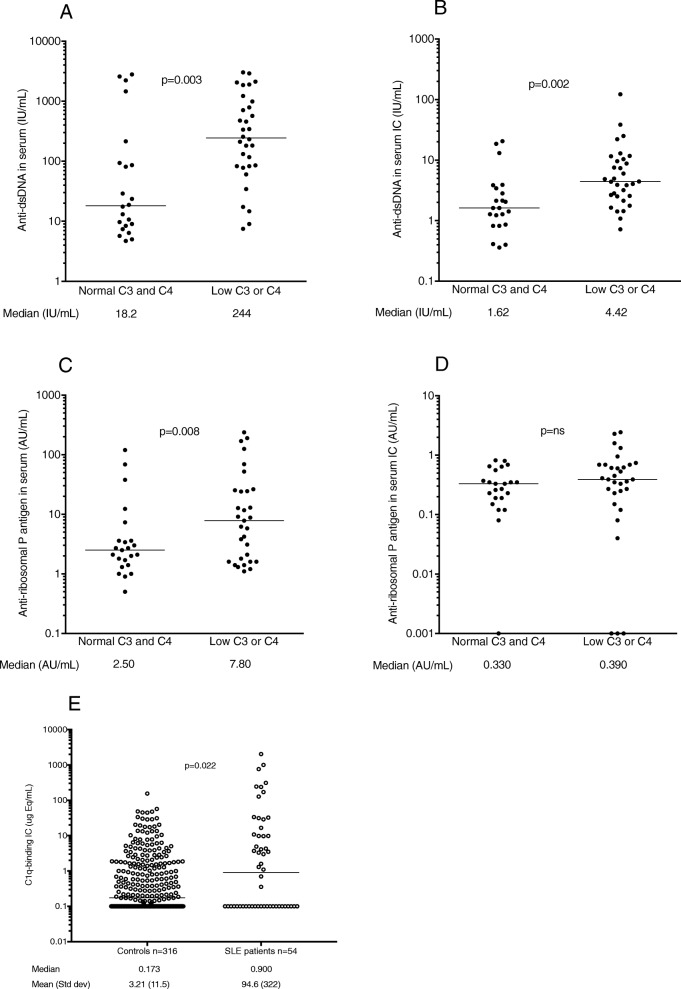
Associations between levels of complement protein C3 and/or C4 and levels of **a**, **b** anti-dsDNA and **c**, **d** anti-ribosomal P antigen antibodies in **a**, **c** sera obtained with conventional measurement and **b**, **d** in corresponding solubilized IC. **e** Levels of C1q-binding IC in 54/55 of the investigated patients, compared to a population-based non-SLE control group. Horizontal lines indicate median levels, with the corresponding figures shown below each group. ns not significant

SLE patients had increased CIC levels compared to controls (Fig. 2g). Most autoantibody levels in sera and solubilized IC except for the anti-SSA/SSB complex correlated to CIC levels, with the highest degree of correlation for anti-dsDNA. When only samples in the positive range were included, correlations only remained significant for anti-dsDNA (Table 3).

**Table 3:** Correlations between levels of C1q-binding IC on the one hand and autoantibody levels in sera or IC on the other hand

	All samples (*n* = 54)		Samples with serum levels > 40 U/mL of the corresponding autoantibody	
Autoantibody	Serum	IC	Serum	IC
dsDNA	*0.756* < 0.001	*0.600* < 0.001	*0.573* < 0.001	*0.490* 0.003
Histone	*0.410* 0.002	*0.454* < 0.001	0.187 0.542	0.460 0.114
Ribosomal P	*0.554* < 0.001	*0.290* 0.034	− 0.571 0.139	0.119 0.779
PCNA	*0.466* < 0.001	0.170 0.219	NA	NA
SSA/Ro52	0.013 0.928	0.025 0.858	0.017 0.942	0.056 0.809
SSA/Ro60	0.055 0.694	− 0.042 0.765	− 0.015 0.937	− 0.314 0.098
SSB/La	− 0.039 0.780	0.096 0.492	0.291 0.415	0.380 0.278
Sm	*0.358* 0.008	*0.291* 0.033	0.064 0.852	0.353 0.287
U1RNP	*0.280* 0.040	*0.270* 0.049	− 0.002 0.995	0.271 0.211
Sm-U1RNP	*0.322* 0.018	0.264 0.054	0.426 0.254	0.460 0.213

Correlations and corresponding *p* values between variables were calculated by Spearman’s *ρ*. Significant correlations are presented in italics. *NA* not applicable (sample size = 1)

### Baseline serum and IC autoantibody levels in relation to disease activity

When patient sera were dichotomized in relation to baseline plasma concentrations of complement proteins C3 and C4 as a measure of serologically active disease, low complement levels were strongly associated with high baseline quantities of anti-dsDNA in both serum (*p* = 0.003; Fig. 2a) and IC (*p* = 0.002; Fig. 2b). A comparable pattern was displayed for levels of anti-ribosomal P antigen in serum (*p* = 0.008; Fig. 2c). IC levels of anti-ribosomal P did however not differ between patients with normal and depressed complement levels (Fig. 2d). No corresponding associations with serum levels of other autoantibodies were found. However, high baseline IC levels of anti-histone (*p* = 0.020), anti-Sm (*p* = 0.043), and anti-Sm-U1RNP (*p* = 0.036) associated with low complement levels (data not shown). In relation to measures of disease activity, IC autoantibody levels expressed as percentages compared to serum or as enrichments in IC yielded no or lower significances as compared to crude autoantibody levels in IC.

Serum levels of anti-dsDNA and anti-Sm at inclusion correlated with the full SLEDAI-2 K score; this was not the case for any other autoantibody specificities in serum or in IC. When changes in antibody levels after belimumab treatment were correlated to corresponding changes in SLEDAI-2 K, decreases of serum levels but not IC levels of antibodies against ribosomal P antigen, PCNA, Sm, U1RNP, and the Sm-U1RNP complex during 2 years of treatment correlated with decreases in SLEDAI-2 K during the same period. A decrease in IC levels of anti-SSA/Ro52 during the first 3 months also correlated with a decrease in SLEDAI-2K (Table 4).

**Table 4:** Correlations between antibody levels in serum (upper part) and IC (lower part) vs. SLEDAI-2K in 55 belimumab-treated SLE patients

	Months	dsDNA	Histone	Rib P	PCNA	SSA/Ro52	SSA/Ro60	SSB/La	Sm	U1 RNP	Sm-U1 RNP
Serum	0	*0.350* (0.010)	0.036 (0.798)	0.230 (0.098)	0.059 (0.675)	− 0.086 (0.541)	− 0.077 (0.582)	− 0.082 (0.561)	*0.272* (0.049)	0.105 (0.454)	0.235 (0.091)
	0–3	0.586 (0.723)	− 0.076 (0.644)	0.223 (0.173)	0.253 (0.120)	0.156 (0.344)	0.326 (0.105)	0.210 (0.200)	0.139 (0.398)	0.264 (0.104)	0.181 (0.272)
	0–6	0.055 (0.776)	0.009 (0.963)	0.206 (0.283)	− 0.253 (0.204)	− 0.128 (0.507)	0.076 (0.696)	− 0.175 (0.364)	0.160 (0.408)	0.003 (0.987)	0.159 (0.409)
	0–12	0.355 (0.089)	0.347 (0.097)	0.029 (0.893)	0.129 (0.549)	0.054 (0.803)	0.277 (0.190)	0.186 (0.386)	− 0.009 (0.968)	− 0.033 (0879)	0.071 (0.742)
	0–24	0.400 (0.081)	0.141 (0.553)	*0.458* (0.042)	*0.542* (0.014)	0.045 (0.850)	0.323 (0.166)	0.276 (0.239)	*0.518* (0.019)	*0.648* (0.002)	*0.565* (0.009)
IC	0	0.139 (0.320)	0.123 (0.379)	− 0.087 (0.535)	− 0.023 (0.870)	0.040 (0.776)	− 0.042 (0.765)	0.103 (0.464)	0.194 (0.164)	0.057 (0.687)	0.129 (0.358)
	0–3	0.002 (0.989)	0.093 (0.575)	0.103 (0.533)	0.105 (0.524)	*0.328* (0.042)	0.040 (0.847)	0.281 (0.084)	− 0.034 (0.839)	0.056 (0.734)	0.153 (0.352)
	0–6	0.037 (0.848)	0.150 (0.439)	0.034 (0.863)	− 0.235 (0.238)	0.187 (0.332)	0.135 (0.485)	0.185 (0.336)	− 0.003 (0.988)	0.209 (0.276)	0.145 (0.452)
	0–12	0.389 (0.060)	0.231 (0.277)	− 0.144 (0.502)	0.112 (0.603)	0.260 (0.221)	0.248 (0.242)	0.285 (0.177)	0.066 (0.760)	0.180 (0.401)	0.137 (0.524)
	0–24	0.200 (0.412)	0.071 (0.774)	− 0.311 (0.195)	− 0.045 (0.857)	0.058 (0.813)	0.138 (0.572)	0.126 (0.608)	0.247 (0.308)	0.128 (0.602)	0.008 (0.976)

SLEDAI-2K values including complement and centrally measured anti-dsDNA were used. In the first rows for each compartment, baseline autoantibody levels are compared with baselined SLEDAI-2K. In the following rows, changes in autoantibody levels in serum or IC during 3-, 6-, 12-, and 24-month follow-up and changes in SLEDAI-2K during the corresponding periods are compared. Spearman’s *ρ* coefficients are shown, with corresponding *p* values within brackets. Significant correlations are depicted in italics. *Rib P* ribosomal P

### Baseline serum and IC autoantibody levels in relation to clinical response to belimumab

**Fig. 3:**
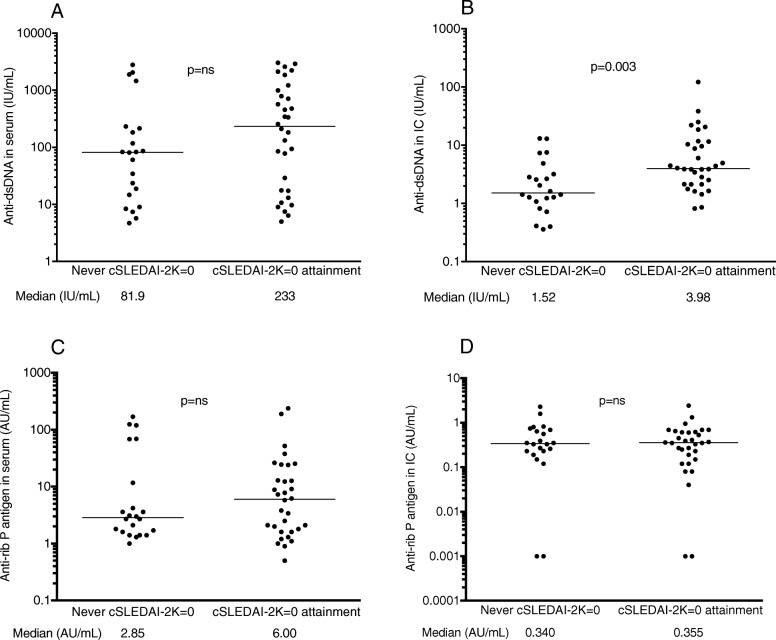
Associations between attainment of cSLEDAI-2K=0 ever during the 2-year follow-up period and levels of **a**, **b** anti-dsDNA and **c**, **d** anti-ribosomal P antigen in **a**, **c** serum obtained with conventional measurement and **b**, **d** in the corresponding solubilized IC. Horizontal lines indicate median values of each antibody, with the corresponding figures shown below each measure. ns not significant, Rib P ribosomal P antigen

No association between baseline levels of anti-dsDNA in serum and attainment of cSLEDAI-2K=0 during follow-up was found (Fig. 3a, also seen previously [29]). On the contrary, high baseline levels of anti-dsDNA in IC were clearly associated with cSLEDAI-2K=0 achievement during the 24-month follow-up (*p* = 0.003; Fig. 3b). Also, high IC levels of anti-histone showed a similar but weaker association with cSLEDAI-2K=0 through follow-up (*p* = 0.048), again with no association with corresponding serum levels. No association was observed between levels of anti-ribosomal P antibodies in serum or IC and cSLEDAI-2K=0 during the follow-up period (Fig. 3c, d). Low baseline levels of anti-Sm and anti-Sm-U1RNP in serum but not in IC were associated with cSLEDAI-2K=0 at month 6 (*p* = 0.018 and 0.040, respectively), and for anti-Sm-U1RNP also after 3 months of treatment (*p* = 0.038). No other antibodies in serum or IC at baseline exhibited any association with cSLEDAI-2K=0 or SRI-4 at individual time points during the follow-up period, except for an association between low IC levels of anti-PCNA and SRI-4 at 3 months of treatment (*p* = 0.025).

**Fig. 4:**
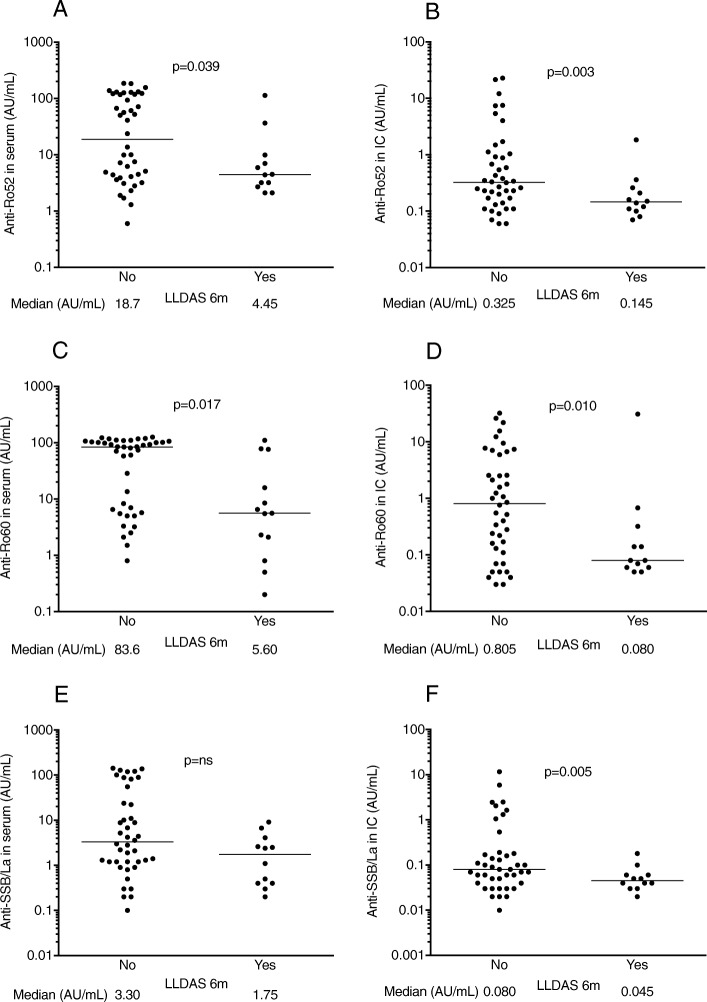
Associations between attainment of LLDAS after 6 months and levels of **a**, **b** anti-SSA/Ro52, **c**, **d** anti-SSA/Ro60, and **e**, **f** anti-SSB/La in **a**, **c**, **e** sera where data were obtained with conventional measurements and **b**, **d**, **f** in the corresponding solubilized IC. Horizontal lines illustrate median levels, with corresponding values presented below each measure. ns not significant, LLDAS low lupus disease activity state

Low levels of antibodies against the SSA/SSB complex were associated with attainment of LLDAS after 6 months of belimumab therapy, and for all these autoantibodies, the associations were stronger with IC levels than with corresponding serum levels (Fig. 4). No association with LLDAS attainment at any occasion was seen for other autoantibodies in sera or IC.

Compared to IC levels as described above, the expression of IC autoantibody levels as percentages of serum levels or as enrichment in IC in comparison to that in serum showed weaker associations to cSLEDAI-2K=0, SRI-4, or LLDAS, or no associations at all (data not shown).

### Changes of autoantibody levels in IC and relation to treatment response

**Fig. 5:**
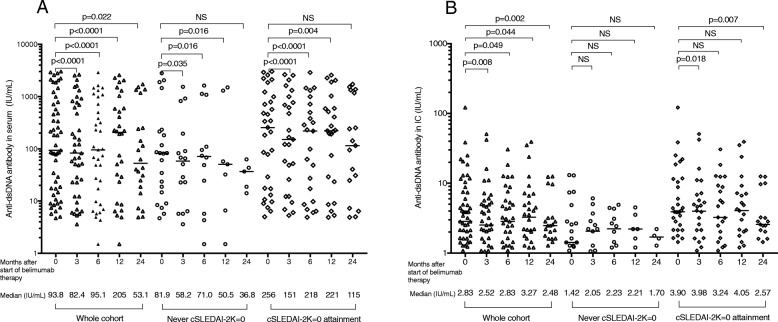
Changes in anti-dsDNA levels **a** in sera where data were obtained with conventional measurements and **b** in solubilized IC for the belimumab-treated SLE patients, in relation to therapy response. In each panel, data are shown for all 53 patients with full data to the left, for the 22 patients not attaining cSLEDAI-2K=0 in the middle, and for the 31 patients attaining cSLEDAI-2K=0 to the right. Horizontal bars represent median values, which are also presented below each measure

Serum levels of the majority of the autoantibodies were reduced already after 3 months of treatment and remained suppressed during the 2-year follow-up period. Levels of anti-dsDNA in IC showed the same pattern of a quick decline, whereas IC levels of the other autoantibodies showed a more gradual decrease, evident only after 12 or 24 months of treatment. After 2 years of follow-up, IC levels of all autoantibodies except anti-SSB and anti-ribosomal P were lower than in baseline sera (Table 5). The decrease in serum anti-dsDNA levels was evident both among patients reaching and not reaching cSLEDAI-2K=0 during the study period (Fig. 5a). In contrast, anti-dsDNA levels in IC decreased only among patients achieving cSLEDAI-2K=0, being significant at months 3 and 24 from baseline (Fig. 5b).

**Table 5:** Percent changes in median (mean) antibody levels in sera and corresponding IC during 24-month follow-up

	0–3 months				0–6 months				0–12 months				0–24 months			
	Serum		IC		Serum		IC		Serum		IC		Serum		IC	
Antibody	Change	*p*	Change	*p*	Change	*p*	Change	*p*	Change	*p*	Change	*p*	Change	*p*	Change	*p*
dsDNA	*− 21.4*	< 0.001	*− 18.2*	0.008	*− 34.4*	< 0.001	*− 25.0*	0.049	*− 32.3*	< 0.001	*− 40.3*	0.044	*− 34.9*	0.022	*− 36.4*	0.002
	(− 17.2)	*n* = 41	(− 6.6)	*n* = 41	(− 27.1)	*n* = 35	(6.2)	*n* = 35	(62.7)	*n* = 30	(1.7)	*n* = 30	(− 24.8)	*n* = 22	(− 28.2)	*n* = 21
Histone	*− 23.1*	< 0.001	0	0.414	*− 26.1*	< 0.001	0	0.687	−24.8	0.1051	−17.9	0.595	−23.6	0.411	*− 13.9*	0.015
	(− 17.2)	*n* = 41	(95.4)	*n* = 41	(5.5)	*n* = 35	(15.6)	*n* = 34	(64.9)	*n* = 30	(23.3)	*n* = 29	(4.4)	*n* = 22	(− 6.5)	*n* = 21
Ribosomal P	*− 20.0*	< 0.001	0	0.993	*− 31.2*	< 0.001	3.33	0.841	*− 36.5*	< 0.001	− 26.9	0.105	*− 41.8*	0.001	− 15.9	0.933
	(− 13.6)	*n* = 41	(49.5)	*n* = 38	(− 32.4)	*n* = 35	(25.2)	*n* = 32	(− 28.7)	*n* = 30	(1.9)	*n* = 28	(− 30.8)	*n* = 22	(40.6)	*n* = 21
PCNA	*−26.0*	< 0.001	−11.2	0.091	*−31.5*	< 0.001	2.6	0.773	*−30.2*	< 0.001	2.2	0.442	*−36.1*	< 0.001	*−31.1*	0.006
	(− 19.0)	*n* = 41	(− 3.0)	*n* = 41	(− 24.6)	*n* = 35	(22.3)	*n* = 35	(− 10.3)	*n* = 30	(15.1)	*n* = 30	(− 35.6)	*n* = 22	(− 17.3)	*n* = 21
SSA/Ro60	*− 2.8*	0.034	− 1.4	0.222	*− 7.1*	0.0115	− 7.1	0.599	*− 7.0*	0.048	− 21.7	0.211	− 6.1	0.0575	*− 33.0*	0.007
	(− 8.6)	*n* = 41	(1.3)	*n* = 41	(− 9.3)	*n* = 35	(30.2)	*n* = 35	(10.2)	*n* = 30	(8.1)	*n* = 30	(28.9)	*n* = 22	(− 6.5)	*n* = 21
SSB/La	*− 11.9*	< 0.001	− 14.3	0.274	*− 14.9*	0.008	− 7.7	0.053	*− 24.9*	0.008	− 20.8	0.051	*− 27.7*	0.041	− 14.2	0.143
	(− 11.0)	*n* = 41	(9.0)	*n* = 41	(− 13.1)	*n* = 35	(16.3)	*n* = 35	(54.2)	*n* = 30	(9.4)	*n* = 30	(− 23.7)	*n* = 22	(23.0)	*n* = 21
SSA/Ro52	− 9.3	0.1787	− 14.3	0.124	− 6.8	0.474	− 3.5	0.315	− 3.8	0.078	− 15.5	0.292	*− 10.6*	< 0.001	*− 28.2*	0.005
	(− 6.8)	*n* = 41	(− 4.7)	*n* = 41	(− 7.7)	*n* = 35	(22.7)	*n* = 35	(− 6.7)	*n* = 30	(8.9)	*n* = 30	(− 20.4)	*n* = 22	(− 11.8)	*n* = 21
Sm	*− 25.0*	< 0.001	0	0.406	*− 16.7*	0.005	0	0.813	− 12.8	0.228	− 27.3	0.292	− 30.0	0.0532	*− 40.9*	0.050
	(− 24.6)	*n* = 41	(0.1)	*n* = 33	(− 18.0)	*n* = 35	(2.0)	*n* = 31	(17.7)	*n* = 30	(− 12.0)	n = 28	(− 1.5)	*n* = 22	(− 26.6)	*n* = 18
U1RNP	*− 13.8*	0.0001	− 12.5	0.111	*− 13.3*	0.010	− 16.7	0.167	*− 12.3*	0.006	*− 26.6*	0.001	*− 22.8*	< 0.001	*− 26.3*	< 0.001
	(− 4.4)	*n* = 41	(4.4)	*n* = 41	(3.4)	*n* = 35	(2.2)	*n* = 35	(17.5)	*n* = 30	(3.8)	*n* = 30	(− 19.3)	*n* = 22	(− 24.4)	*n* = 21
Sm-U1RNP	*− 14.7*	< 0.001	0	0.621	*− 15.2*	< 0.001	− 14.3	0.137	*− 22.4*	0.009	*− 33.2*	0.019	*− 31.5*	< 0.001	*− 25.0*	0.007
	(− 17.4)	*n* = 41	(7.4)	*n* = 40	(− 15.7)	*n* = 35	(0.8)	*n* = 34	(11.1)	*n* = 30	(4.9)	*n* = 30	(− 25.5)	*n* = 22	(− 9.2)	*n* = 21

Differences were compared pairwise with the Wilcoxon signed rank test. Negative values indicate decreasing antibody levels; significant changes are presented in italics. The number of SLE-samples in the individual pairwise comparisons is stated below the *p* values

## Discussion

Baseline levels of anti-dsDNA in IC but not in serum were highly associated with clinical response to belimumab therapy. Low IC levels of anti-SSA/SSB associated with attainment of LLDAS, a composite tool developed to reflect a low SLE disease activity state [38, 40]. Interestingly, this association was stronger for IC levels than for the corresponding serum levels. The findings are in agreement with a recent study showing no association between serum autoantibody levels and attainment of LLDAS after belimumab therapy and collectively argue that measurement of autoantibody levels in the IC fraction of serum may better associate with clinical response to belimumab treatment, as compared to the corresponding autoantibody levels in serum [41, 42].

Baseline serum levels of both anti-dsDNA and anti-ribosomal P associated with low complement levels, but levels in IC showed different associations. Whereas high baseline quantities of anti-dsDNA in IC were strongly associated with low complement levels, this was not seen for antibodies against the anti-ribosomal P antigen. Together, these findings imply that measurement of autoantibody levels in IC yields different associations both to SLE disease activity as to clinical response to belimumab therapy, compared to conventional measurement of autoantibody levels in serum.

Our findings are consistent with previous studies showing that patients with elevated serum anti-dsDNA levels prior to treatment initiation are expected to show a favorable response to belimumab treatment compared with placebo [27]. However, the present data suggest that levels of anti-dsDNA in IC may prove even more useful for predicting treatment outcome. This hypothesis is corroborated by the fact that diminished IC levels of anti-dsDNA were found only among patients attaining SLEDAI-2K=0 during the follow-up period.

In contrast, no association with SRI-4 was found. From a clinical point of view, however, clinical remission (here defined as cSLEDAI-2K=0) and low disease activity state (here defined as LLDAS) may be considered more relevant treatment outcomes compared with reduction of SLE activity based on SRI-4 which may be achieved despite persisting moderate/high disease activity in patients with highly active disease at treatment initiation [43].

Median levels of anti-dsDNA in IC were 2.3% of those in serum, and the corresponding figures were 0.8–13% for the investigated autoantibody specificities, all of which were enriched in IC. The strongest associations to clinical remission, here defined as a cSLEDAI-2K score of zero attained during the follow-up period, were seen for actual levels of autoantibodies in IC. Levels expressed as percentages of serum levels with or without correction for total IgG levels weakened these associations. These other calculations were made as autoantibody quantification in IC is a new field without any previous knowledge concerning how to express data in an informative way from a clinical perspective. So far, measurement of autoantibody levels in IC without correction for levels in serum or levels of total IgG seems to be the measure most closely associated with disease activity and response to belimumab treatment in SLE patients.

It is still not clarified whether anti-dsDNA mainly deposit in target organs as monovalent antibodies or as pre-formed circulating IC. Our results argue that diminished passive entrapment of pre-formed circulating IC might be of importance in SLE patients who show good responses to belimumab therapy. Alternatively, the anti-dsDNA fraction detected in circulating IC might co-vary with tissue sequestration because both fractions share propensities, e.g., concerning cationic charge, but without actual sequestration of IC-bound autoantibodies. Except anti-dsDNA, autoantibodies against Sm, SSA and SSB, C1q, and C-reactive protein (CRP) have been described in glomerular immune deposits in lupus nephritis patients [11, 44]. These results are in agreement with our finding of low LLDAS in patients with high IC levels of anti-SSA/SSB and argue that belimumab therapy may decrease the levels of different autoantibodies in circulating IC.

Previous studies on IC levels of anti-dsDNA used ultracentrifugation, a time-consuming technique that can only investigate a limited number of samples in parallel [6]. Ultracentrifugation results did not correlate with autoantibody levels in IC obtained with PEG precipitation, a commonly used IC separation technique known to also precipitate a wide array of high molecular weight proteins along with IC [45]. We believe that our new technique combines purity of obtained IC eluates with a capacity to handle large numbers of samples in clinical studies.

Early studies showed an association between persistence of circulating DNA-containing IC and increased morbidity and resistance to treatment of SLE patients. Disappearance of DNA-containing IC associated with clinical remission and improvement in glomerular filtration rate and central nervous system disease [12]. In agreement with this, we found decreased IC levels of anti-dsDNA in clinical responders to belimumab therapy, but not in non-responders.

Only some anti-dsDNA antibodies form circulating IC. An important question to resolve is whether the fraction of anti-dsDNA participating in the formation of such circulating IC share characteristics with pathogenic anti-dsDNA, e.g., concerning the degree of cross-reactivity and cationic charge. Our technique can be used to study whether anti-dsDNA found in IC and in monovalent form in the circulation differ in such respects.

Limitations of this study include the rather low number of study subjects, and quantification of very low levels of autoantibodies in IC, below levels considered significant in serum samples. We also do not know whether certain autoantibody levels in IC should be used as thresholds for positivity. Twenty blood donors were investigated in parallel to the patients. Although these controls expressed far lower levels of antibodies both in sera and IC compared to the lupus patients they were too few to establish meaningful cutoffs, and we found that evaluation of quantitative levels to be more valid in the present study.

## Conclusion

By quantifying specific autoantibodies in circulating IC, we were able to demonstrate that IC levels of autoantibodies against dsDNA and the SSA/SSB complex were associated with treatment response in belimumab-treated SLE patients. We envisage that in the future, this methodology might be used to identify patients who are expected to respond to treatment with belimumab and other therapies for SLE and other IC-associated diseases, as diagnostic tools, and to characterize the unique properties of the fraction of autoantibodies that participates in the formation of circulating IC.

## Data Availability

The datasets supporting the conclusions of this article are included within the article.

## Abbreviations

ACPA: Anti-citrullinated peptide antibodies
ALBIA: Addressable laser bead immunoassay
ANA: Anti-nuclear antibody(ies)
AU: Arbitrary unit(s)
BAFF: B cell activating factor
BLyS: B lymphocyte stimulator
CIC: Circulating C1q-binding immune complex(es)
CRP: C-reactive protein
cSLEDAI-2K: Clinical systemic lupus erythematosus disease activity index 2000
DNA: Deoxyribonucleic acid
dsDNA: Double-stranded deoxyribonucleic acid
ELISA: Enzyme-linked immunosorbent assay
IC: Immune complex(es)
IU: International unit(s)
LLDAS: Low lupus disease activity state
PBS: Phosphate-buffered saline
PCNA: Proliferating cell nuclear antigen
Rib P: Ribosomal P antigen
RT: Room temperature
SRI-4: SLE Responder Index 4
SSA: Sjögren syndrome antigen A
SSB: Sjögren syndrome antigen B
SLE: Systemic lupus erythematosus
SLEDAI-2K: Systemic lupus erythematosus disease activity index 2000
SLICC: Systemic Lupus International Collaborating Clinics
Sm: Smith antigen
U1RNP: U1 small nuclear ribonucleoprotein
